# A blinded, randomized and controlled multicenter field study investigating the safety and efficacy of long-term use of enflicoxib in the treatment of naturally occurring osteoarthritis in client-owned dogs

**DOI:** 10.3389/fvets.2024.1349901

**Published:** 2024-02-23

**Authors:** Josep Homedes, Marion Ocak, Sebastian Riedle, Marta Salichs

**Affiliations:** ^1^Ecuphar Veterinaria SLU (Animalcare Group) Sant Cugat del Vallès, Barcelona, Spain; ^2^MD Research, Pullach i. Isartal, Germany; ^3^Conreso GmbH, Munich, Germany

**Keywords:** dogs, non-steroidal anti-inflammatory drugs, osteoarthritis, pain—drug therapy, enflicoxib

## Abstract

**Background:**

Enflicoxib is a COX-2 selective NSAID shown to be efficacious and safe in the treatment of pain and inflammation associated with canine osteoarthritis (OA) in clinical studies of 6 weeks duration.

**Objective:**

This prospective, multisite, blinded, randomized, placebo-controlled, parallel-group field study aimed to confirm the safety and efficacy of enflicoxib in long-term canine OA treatments.

**Animals:**

A total of 109 client owned dogs with clinical and radiographic signs of OA for at least 3 weeks were enrolled with 78 dogs completing all study visits.

**Methods:**

Dogs were randomized at a 3:1 ratio to receive enflicoxib (*n* = 83) or placebo (*n* = 26) once weekly during 6 months. Dogs underwent veterinary assessments from Day 0 to Day 189 using a clinical sum score (CSS). Efficacy was also assessed by the owners using the Canine Brief Pain Inventory (CBPI). Safety was assessed clinically and by repeated blood and urine sample analysis. The efficacy outcome measure was the treatment response according to the CSS and secondarily the treatment response according to the CBPI. The primary safety outcome was the incidence of adverse events (AEs) and secondarily the evolution of the clinical pathology parameters.

**Results:**

Percentages of CSS responders for enflicoxib were 71.6; 74.6 and 71.6% on Days 44, 135 and 189 respectively, always showing statistically significant differences (*p* < 0.05) vs. placebo (41.7, 33.3, and 20.8% respectively). Treatment response according to owner assessments followed the same pattern, achieving significant differences compared to placebo after 2 weeks of treatment. The incidence and type of AEs were as described in previous enflicoxib studies of shorter duration and as for other NSAIDs, with no tendency to increase over time. No relevant changes in hematology, biochemistry or urine parameters were observed.

**Conclusions and clinical relevance:**

Enflicoxib safety and efficacy profile is maintained after a long-term treatment, which together with its weekly administration, makes it a good alternative for the chronic treatment of dogs with naturally occurring OA.

## 1 Introduction

Canine osteoarthritis (OA) is a multi-factorial, progressive, degenerative disease of synovial joints that leads to chronic pain, loss of joint function and impaired mobility. It is highly prevalent within the dog population, with substantial implications for their quality of life and welfare (1).

Best practice to treat dogs with OA is to follow a multimodal therapy program, and non-steroidal anti-inflammatory drugs (NSAIDs) are recommended at first-line therapy for management of the associated pain and inflammation (2–4).

Enflicoxib, a recently launched NSAID of the coxib class, is dosed at weekly intervals, with an initial loading dose of 8 mg/kg, followed by once weekly doses of 4 mg/kg (Daxocox^®^ tablets for dogs, Ecuphar/Animalcare group) (5). Enflicoxib has shown to be efficacious and safe in various laboratory models of pain and inflammation (6), in arthritis models in dogs (7), and in randomized field clinical studies in dogs with naturally occurring OA over a 6-week treatment period (8, 9).

As with most clinical trials assessing the efficacy and safety of NSAIDs in dogs, these field clinical studies with enflicoxib demonstrated its efficacy and safety for a relatively short period. However, considering the chronic nature of canine OA and the current recommendations to prescribe sustained long-term treatments to prevent central sensitization (2), it is necessary to guarantee that long periods of continuous treatment would also be well tolerated under field conditions. Indeed, a previously published systematic review highlighted the lack of long-term clinical trials of NSAIDs (10). In a controlled overdose laboratory study with Beagle dogs, enflicoxib has shown to be very well tolerated with a broad safety margin up to five times the recommended dose for 3 months and at three times the recommended dose for 7 months (11). However, in dogs treated with NSAIDs, adverse events (AEs) appear to be more commonly reported in clinical trials when compared with research studies, as these use young healthy animals, in contrast to clinical trials where older dogs with naturally occurring OA with comorbidities and concomitant treatments are enrolled (12, 13).

Therefore, although it has been described that long-term use of NSAIDS in dogs with OA is not associated to an increased risk of AEs (10), it is necessary to confirm that the good safety profile seen for enflicoxib in the long-term overdose study is maintained when enflicoxib is used for long periods in the “real world” target population.

The aim of this field clinical trial was to evaluate the safety of enflicoxib, and to confirm its superior clinical efficacy compared to a negative control group, when administered at the recommended dose, and for the first time, for a period of 6 months, for the treatment of pain and inflammation associated with OA in client-owned dogs.

## 2 Materials and methods

### 2.1 Study design

This study was a prospective, multisite, blinded, randomized, superiority, placebo-controlled, parallel-group field study conducted in compliance with the Veterinary International Conference on Harmonization guideline for Good Clinical Practice (14). The study was performed at 7 veterinary practices located in various regions of Hungary (*n* = 5) and Portugal (*n* = 2). Recruitment period run from September 2020 to March 2021. Last follow up visit was in August 2021 (total study duration was 330 days).

The protocol was finalized *a priori* and satisfied national legislation and animal welfare requirements. Approval was obtained from the Portuguese and Hungarian regulatory authorities: DGAV (Direção Geral de Alimentação e veterinária) and NEBH (Nemzeti Élelmiszerlánc-biztonsági Hivatal), with authorization numbers 82/ECVPT/2020 and 5300/3265-2/2020, respectively. Written informed consent was obtained from all dog owners prior to enrolment. Dogs remained under the care of their owners at home during and after the study.

Owners had to accept adherence to the study visits and the given dosing instructions, were instructed to report any unusual effect on the dog and were taught not to change, as far as possible, the daily exercise routine or home management of their dogs during the study in order not to have an impact on the evaluation of efficacy of the test product.

### 2.2 Animal selection

All dogs were client-owned and presented as patients at the veterinary practices. Dogs older than 6 months of any breed and sex could be enrolled in the study. On first examination, dogs were required to have clinical signs of OA (pain and lameness) for at least 3 weeks along with radiographic evidence of OA (presence of articular lesions compatible with OA, such as subchondral bone sclerosis, bone remodeling, osteophytes, irregular or diminished joint space) in at least one joint of the pelvic or thoracic limbs.

At the initial visit, dogs were evaluated by the veterinarian for possible inclusion in the study. General history was recorded, and dogs underwent a general physical examination and blood samples were taken for hematology and biochemistry. Urine samples were also taken for test stick and urine density.

Further to the exclusion criteria related to non-permitted anti-inflammatory and analgesic previous medication or disease modifying and chondroprotective agents or diets as well as concomitant diseases and the concomitant treatment administration restrictions included in previous efficacy clinical trials with enflicoxib (8, 9), dogs could not have received intra-articular injections of any type for 1 year and depot anti-inflammatory drugs for at least 120 days.

Several reasons for withdrawal of dogs from the study were applicable as previously described (8, 9). For cases withdrawn due to worsening of clinical signs of OA or unsatisfactory therapeutic response, additional veterinary care including rescue therapy was permitted after withdrawal of the dog from the study. A rescue therapy was the use of a prohibited product indicated for the treatment of OA. A forbidden concomitant treatment was a therapy that could interfere with the assessment of pain for which the indication was not related to OA.

### 2.3 Assessments

On the day of inclusion and before first treatment administration, the severity of clinical signs of OA was evaluated by both, the veterinarian, and the owner.

The veterinarians assessed pain and lameness using previously described numerical rating scales (NRS). This NRS included the assessment of three parameters: General Musculoskeletal Condition, Lameness/Weight Bearing and Pain on Palpation/Manipulation of Joint(s). Each parameter was scored with a severity grade from 0 (clinically normal) to 4 (nearly incapacitated). The clinical sum score (CSS) was the sum of scores for these three parameters and ranged from 0 to 12 (15) (see Supplementary material).

Owners were instructed by the veterinarian to assess their dogs in their home environment. To assure consistency among owners and over time, a validated scale was used to describe dogs pain severity, pain interference and the overall impression of quality of life (QoL) according to the Canine Brief Pain Inventory (CBPI) questionnaire (16, 17), considering the condition of the dog over the previous 7 days. The pain severity score (PSS) is the arithmetic mean of 4 items scored on an 11-point (0–10) numerical scale, and the pain interference score (PIS) is the mean of 6 items scored similarly (0 = no pain or interference and 10 = severe pain or interference). The overall impression of the dog’s QoL was rated in absolute categories as poor, fair, good, very good or excellent.

To be eligible for inclusion in the study a basal CSS ≥ 4 and a PSS and PIS scores ≥2 was required on Day 0, prior to treatment. Although some dogs could have mild and well controlled health conditions unrelated to OA, dogs would be required to be in good general health based on a complete general physical examination, and satisfactory blood (hematology and biochemistry) and urine examination results.

### 2.4 Randomization and blinding

Dogs meeting all inclusion criteria and none of the exclusion criteria were enrolled by the veterinarian. Afterwards, the dispenser allocated each dog to the enflicoxib or the placebo groups by use of a randomized block schedule generated by the statistician at the predefined enflicoxib:placebo ratio of 3:1. Dogs in the enflicoxib group received Daxocox^®^ tablets for dogs (Ecuphar/Animalcare group), while dogs in the placebo group received the same formulation without the active ingredient. Products were provided as ready to use tablets in anonymized blisters and boxes. The random allocation was implemented using sequentially coded boxes following a randomization list provided at each site. Dispensers were responsible for the preparation and dispensing of study treatments as well as treatment accountability. All forms related to treatment and the randomization list were stored in secured location by the dispenser. The veterinarian, or any other personnel involved in clinical evaluations, as well as the owners remained blinded to treatment. Day 0 was defined as the day of inclusion and the first day of treatment for each dog.

### 2.5 Treatments

Dogs allocated to the enflicoxib group received an initial oral loading dose of 8 mg/kg on Day 0, and subsequent once weekly (±2 days) maintenance oral doses of 4 mg/kg, for 25 additional weeks. Dogs allocated to the placebo group received placebo tablets under the same dose regimen (number of tablets) to mimic enflicoxib posology. The dispenser administered the loading dose on Day 0, all subsequent doses were administered by the owner at home.

Dose calculations for study treatments were performed using the body weight determined on Day 0 and following label dosing instructions (5). The number of tablets was adjusted after each veterinary visit, if needed. As food increases absorption and following label indications, enflicoxib or placebo tablets were administered with food or immediately before feeding.

### 2.6 Efficacy assessments

Following the initial veterinary assessment on Day 0 (prior to treatment), dogs were re-evaluated on Days 44 (±2 days), 135 (±7 days) and 189 (±7 days) at the veterinary practice. Phone calls to the owners were performed on Days 7 (±2 days), 14 (±2 days), and 90 (±4 days).

During each visit, general physical examinations and clinical assessments of pain and lameness using the CSS were performed by the veterinarian. The most severely affected joint was selected on Day 0, prior to the start of treatment administration, and evaluated throughout the study regardless of whether another joint was also affected. Dogs were evaluated while walking and trotting and their gait was assessed while turning in a tight circle or while going up and down stairs. After completion of the evaluations at exercise, each dog was observed while standing for signs of weakness, asymmetric limb trembling, spasms, and asymmetry of limb carriage or weight bearing, including elevation of limbs contra-lateral to those affected to assess the degree of resistance.

In addition, during each visit the veterinarian interviewed the owner to record their assessments using the CBPI. Additionally, CBPI was also recorded during the phone contacts. The owner was not aware of the required threshold level for PSS and PIS scores for inclusion in the study and did not have access to the scores of previous assessments when completing each CBPI.

### 2.7 Efficacy outcome measures

The primary efficacy criterion was the response to treatment (successful ‘overall improvement’) based on the veterinary assessment using the CSS. A dog was classified as a responder when at least two of the three parameters of the veterinary assessment improved compared to Day 0. The changes of the CSS scores compared to Day 0 were also calculated and summarized by treatment and assessment day.

The secondary efficacy criterion was the response to treatment (successful ‘overall improvement’) based on the owner assessment using the CBPI. A dog was classified as a responder if it had a decrease ≥1 in PSS, and ≥2 in PIS compared to basal scores on Day 0 (18, 19).

For the QoL assessment, the percentage of dogs improving in at least one absolute category compared to Day 0 was calculated and compared between groups at each time point.

In case of excessive pain, dogs could be withdrawn from the study by the veterinarian and receive rescue treatment. Any dog withdrawn from the study due to administration of rescue treatment, lack of efficacy or inadequate improvement was considered a non-responder.

### 2.8 Safety outcome measures

Blood samples for hematology and biochemistry as well as urine samples were collected on Day 0 (prior to treatment), Day 44 and Day 189.

The following hematological and biochemical parameters were determined: red blood cell (RBC) count, white blood cell (WBC) total and differential count, platelet (thrombocyte) count and estimate, hematocrit and hemoglobin and reticulocytes. Amylase, albumin, alkaline phosphatase, alanine-aminotransferase (ALT), aspartate-aminotransferase (AST), calcium, cholesterol, creatinine, creatine kinase, globulin, glucose, γ-Glutamyltransferase (γ-GT), magnesium, phosphorus, potassium, sodium, total bilirubin, total protein, and urea.

Urine samples were analyzed for blood/erythrocytes/hemoglobin, glucose, ketone bodies, protein, leukocytes, nitrite, specific weight, pH-value, bilirubin, and urobilinogen.

Safety was also assessed by recording any AEs that occurred during the study irrespective of nature and severity or whether to be product related or not. Safety criteria included the clinical signs, severity, causality evaluation and incidence calculations. Adverse events description, recording and assessment was performed as previously described (8, 9) and following the Veterinary Dictionary for Drug Regulatory Activities (VeDDRA) terms (20) and the ABON system of causality assessment (20). This assessment considered that NSAIDs have the potential to cause or exacerbate gastrointestinal, renal, and hepatic disorders. Events related to suspected lack of efficacy were also included.

For the calculation of the incidence, when several AEs were observed in a single animal at an overlapped time frame, according to current guidelines, they were considered as different clinical signs of the same AE.

### 2.9 Data handling

All data were collected on paper data capture forms by the participating investigators at each study site. The investigators were instructed to send the completed forms by fax, scan, or picture to the study monitor as soon as each form had been completed. Raw Data (forms etc.) were stored at the study sites until collected by the study monitor. Apart from on-site visits, the study monitor had regular contacts (as needed) with the investigators by email and telephone. Quality checks were performed, and once all queries were solved, a double data entry was performed in a Microsoft Access database. After verification of the accuracy of the database, the database was soft locked and audited. After the quality audit, the database was hard-locked, downloaded to Microsoft Excel spreadsheets and transferred to the Statistician responsible for data analysis.

### 2.10 Sample size

The sample size was calculated for the comparison of the treated group to a placebo group with respect to the primary efficacy endpoint. As the protocol included a group of dogs that would not receive any analgesic treatment, and despite the veterinarian could withdraw from the study, at any time, any animal showing excessive pain to maintain animal welfare as much as possible, it was decided to reduce the size of the placebo group to the minimum that allowed a reliable statistical comparison. Therefore, the number of dogs in the placebo group was calculated to be one third of the enflicoxib group.

From the efficacy point of view, considering that previous experience describes a placebo effect of 30% (8, 9), and the desired proportion 3:1 of dogs in enflicoxib and placebo groups, a sample size of 60 and 20 dogs would be required, respectively. This sample size would provide 90% power to detect differences in the primary efficacy endpoint with a 0.05 two-sided significance level (Chi-Square test). Attending to the extended duration of the study, a high incidence of dropouts was expected before the last visit of Day 189. Therefore, final sample size was increased to 75 dogs in the enflicoxib group and 25 in the placebo group.

From the safety point of view, if all enrolled animals were useful for safety assessment, this sample size would provide a probability greater than 95% to observe AEs with an incidence greater than 4%.

Sample size calculations have been performed using SAS System^®^ v9.4 (SAS Institute Inc., Cary, NC, United States).

### 2.11 Statistical analysis

The analysis of the efficacy parameters was performed with the validated program Report Version 6.7 from IDV Datenanalyse und Versuchsplanung, Gauting, Germany (validation of software, hardware, and user according to FDA 21 CFR Part 11). Safety parameters were analyzed with the program SigmaPlot Version 13.0 from Systat Software, San Jose, CA, United States.

Demographic and baseline data evaluation was carried out on all enrolled animals to confirm the balanced distribution of dogs in the two groups. Study deviations were blindly evaluated in order to assign each case to the corresponding population. The statistical analysis for efficacy was performed with the Per Protocol (PP) Efficacy population that included all animals that were randomized and presented at least one post-baseline efficacy data on Day 44, except cases with major deviations that would affect the results. The treatment response classification for the veterinary and owner assessments, as well as the CSS scores at the time of withdrawal from the study were carried forward to all subsequent time points subjected to the Last Observation Carried Forward (LOCF).

For the analysis of safety parameters all enrolled animals that received at least one dose of the test product or placebo were included in the Intent-To-Treat (ITT) population.

Differences between groups for quantitative variables were analyzed by means of Wilcoxon tests. For ordinary scaled data Mantel–Haenszel tests and for categorical variables, differences between groups were evaluated by means of the appropriate test (Chi-Square test or Fischer’s exact test).

All statistical tests were performed two-sided at an overall 5% (*p* < 0.05) level of significance. No multiple testing procedure was applied, therefore all *p*-values—except for the primary criterion must be interpreted descriptively.

## 3 Results

One hundred and nine dogs with clinical and radiographic signs of OA were enrolled and included in the ITT population (83 in the enflicoxib group and 26 in the placebo group). Mean bodyweight (bw) of animals on Day 0 was 27.29 kg (±11.74) and it ranged from 5 to 63 kg and the mean age was 9.30 years ranging from 11 months to 17 years. Males and females, entire or neutered were enrolled in each treatment group and dogs were predominantly purebred. The affected joints included the hip joint (45 [41%]), elbow joint (28 [26%]), stifle joint (28 [26%]) and other joints (8 [7%]). Demographic data is summarized in Table 1.

**Table 1:** Demographic data and CSS, PSS, and PIS basal scores for the ITT population.

	Enflicoxib	Placebo
	*n* = 83	*n* = 26
**Sex, *n* (%)**		
Male	48 (57.8%)	13 (50.0%)
Female	35 (42.2%)	13 (50.0%)
**Age, years**		
Mean (SD)	8.75 (3.10)	9.19 (3.56)
Range	2–16	1–14
**Bodyweight, kg**		
Mean (SD)	27.03 (14.68)	21.49 (11.10)
Range	5–65	4–41
**Breed, *n* (%)**		
Mongrel	41 (49.4%)	13 (50.0%)
Purebred	42 (50.6%)	13 (50.0%)
**Affected joint**		
Hip	34 (41.0%)	11 (42.3%)
Elbow	23 (27.7%)	5 (19.2%)
Stifle	18 (21.7%)	10 (38,5%)
Shoulder	2 (2.4%)	0 (0.0%)
Other^†^	6 (7.2%)	0 (0.0%)
CSS*		
Mean (SD)	7.46 (1.91)	6.46 (1.48)
Range	4–12	4–9
**PSS**		
Mean (SD)	5.14 (1.89)	4.51 (0.97)
Range	1–9	2.3–6.5
**PIS**		
Mean (SD)	5.26 (2.11)	4.63 (1.76)
Range	0.7–9.8	0.3–7.8

*p < 0.05.

^†^These were specified as carpus (2x), hock, metatarsophalangeal joint, radiocarpal joint, and tarsus.

Out of this population, 18 animals were withdrawn before the first visit on Day 44, which resulted in a PP Efficacy population of 91 dogs (67 in the enflicoxib group and 24 in the placebo group). Thirteen dogs were withdrawn due to the late notice that the inclusion criteria were not met (four had an abnormal basal laboratory value and nine did not comply with the minimum CBPI scores), three due to owner’s decision, one with a major protocol deviation, and one experienced an AE. These dogs remained in the safety database as they received, at least, one enflicoxib or placebo dose.

**Figure 1:**
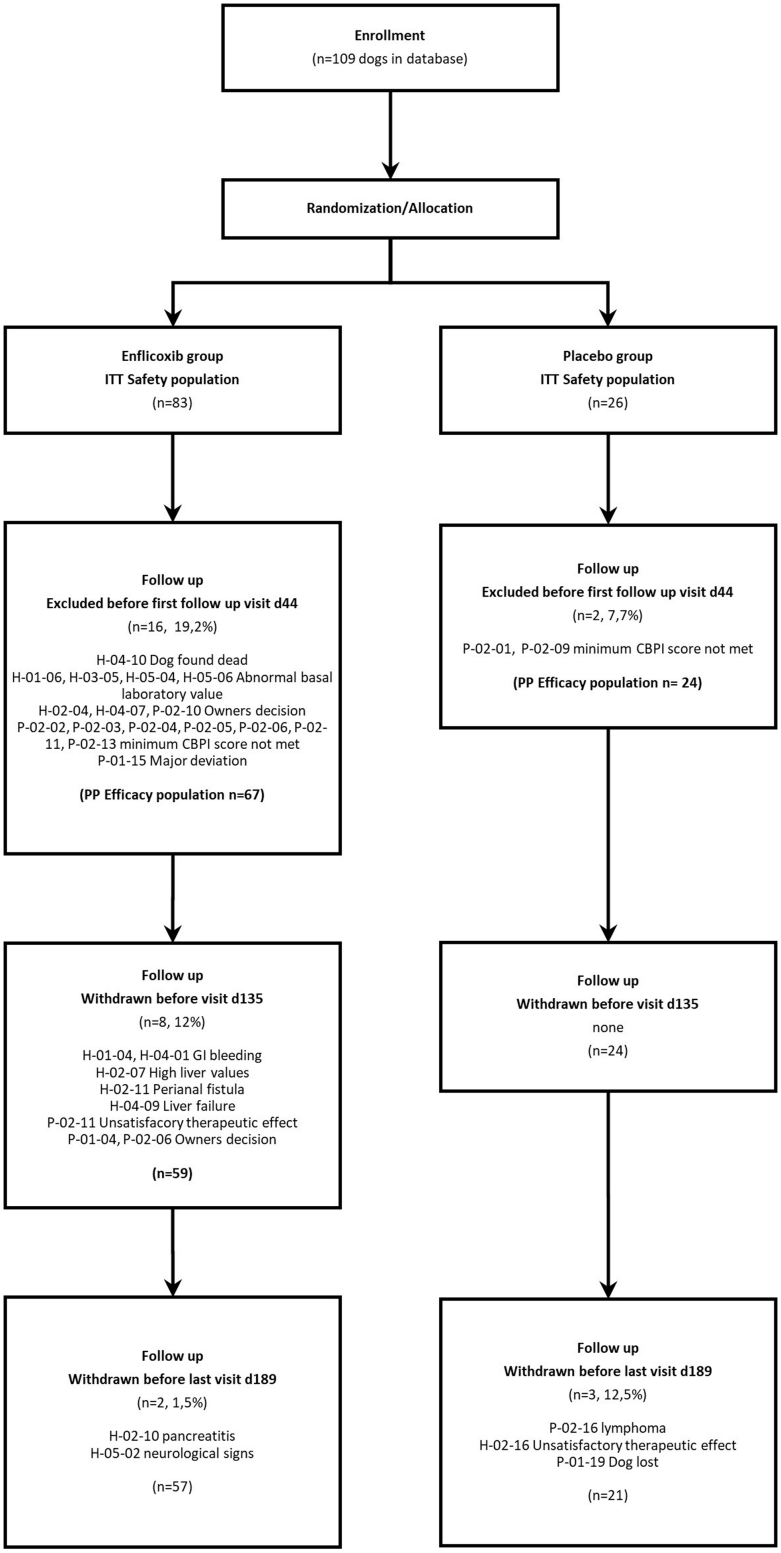
Flowchart showing number of patients recruited, allocated to each treatment, and analyzed.

After the Day 44 visit, two dogs were withdrawn due to owner’s decision, two dogs due to an unsatisfactory therapeutic effect, one dog was lost, and eight experienced some sort of AE (related to treatment or not). The number of dogs remaining in each visit were 91 on Day 44, 83 on Day 135 and 78 on Day 189. See Flowchart in Figure 1.

Twenty-three dogs received a variety of medications that were administered concurrently with either enflicoxib (17) or placebo (5) during the study. The types of medications included anthelmintics, antimicrobials, antiepileptics (phenobarbital), diuretics and pimobendan to treat heart conditions, levothyroxine for a hypothyroid dog and gastric protectants, probiotics, vitamins, benzodiazepines, or sedative products to treat the disorders observed in some AEs reported during the study. No apparent interactions were observed between these medications and enflicoxib or placebo.

### 3.1 Efficacy evaluation

Average CSS decreased with enflicoxib treatment from the first follow up visit on Day 44 (average reduction of 39.7%) and remained lower compared to Day 0 until the end of the study on Day 189.

The average CSS reduction in the placebo group was smaller but noticeable on Days 44 and 135 (average reduction of 21.4% at both time points) compared to Day 0. However, after Day 135 average CSS increased, and the reduction observed at the last visit on Day 189 was only of 10.1% compared to Day 0.

**Figure 2:**
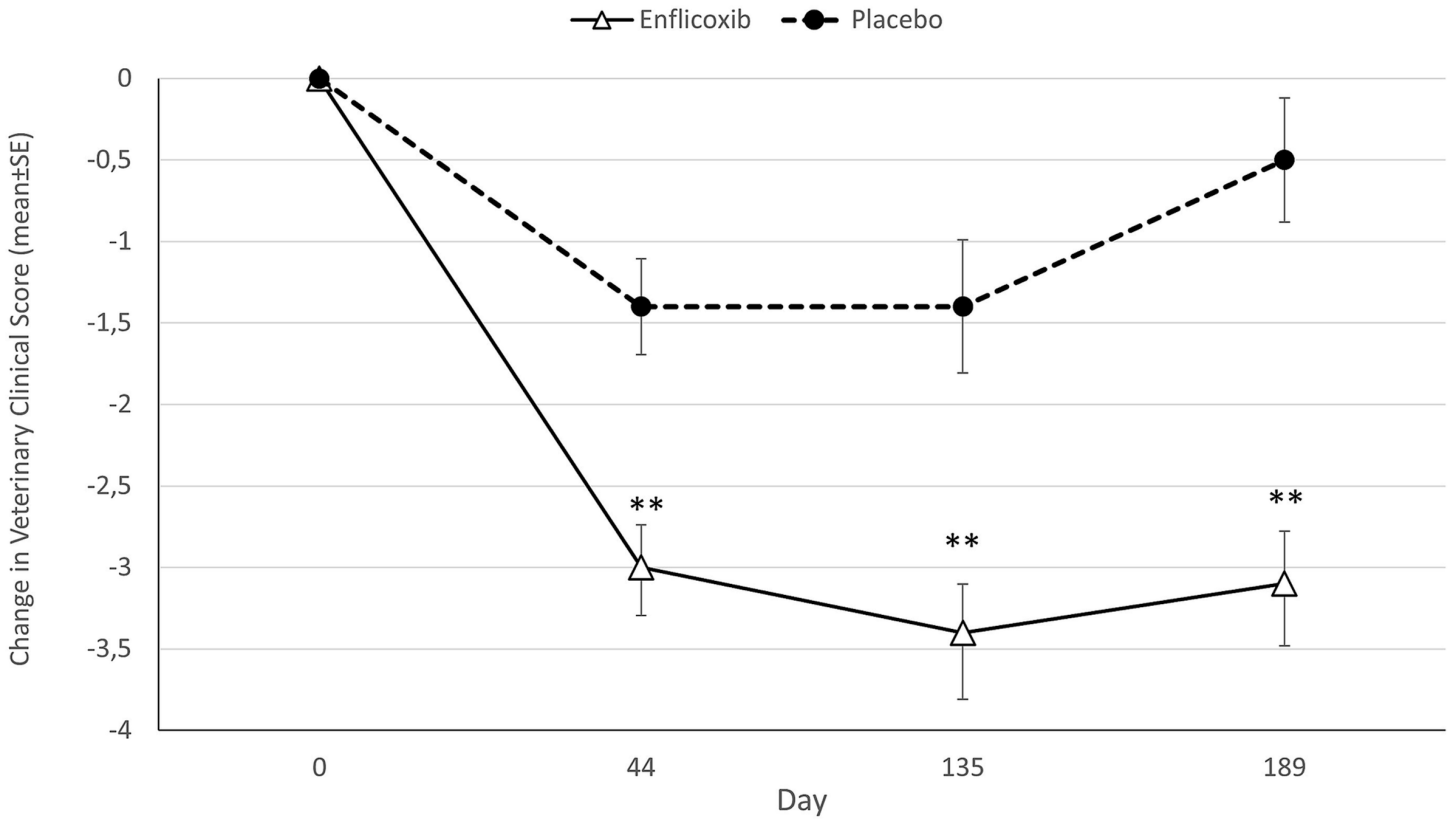
Differences in average CSS (mean ± standard error) for each timepoint and treatment compared to Day 0. Asterisks indicate statistically significant differences of enflicoxib vs. placebo (**p < 0.01).

The reduction of the average CSS in the enflicoxib group was significantly higher in comparison with the placebo group at all follow up visits (*p* < 0.01). The evolution in the decrease over time of the CSS in both groups is shown in Figure 2.

**Figure 3:**
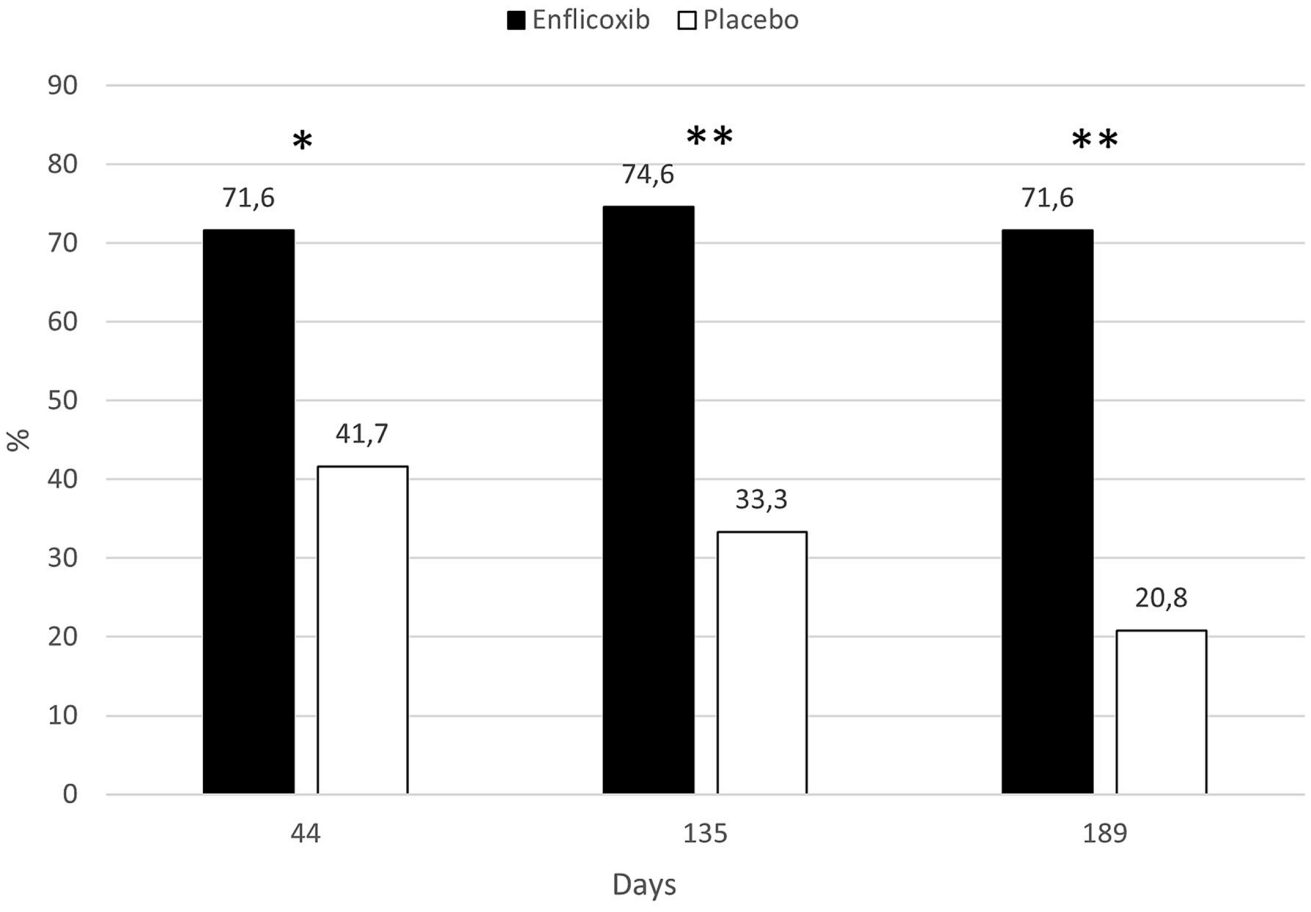
Percentage of CSS responders in each treatment group and time point during the study. Asterisks indicate superiority vs. placebo (*p < 0.05, **p < 0.01).

According to the CSS responder criteria, 71.6% of dogs treated with enflicoxib responded to treatment at the first follow up visit on Day 44, and the percentage of responders remained stable until the last visit. In the placebo group, 41.7% of the dogs were classified as responders in the first follow up visit on Day 44. However, the percentage of treatment response decreased progressively until the 20.8% observed at the last visit on Day 189. The percentage of responders in the enflicoxib group was significantly higher at all visits compared to the placebo group (*p* < 0.05 for Day 44; *p* < 0.01 for Days 135 and 189). Figure 3 shows the treatment response rate according to the CSS throughout the study.

**Figure 4:**
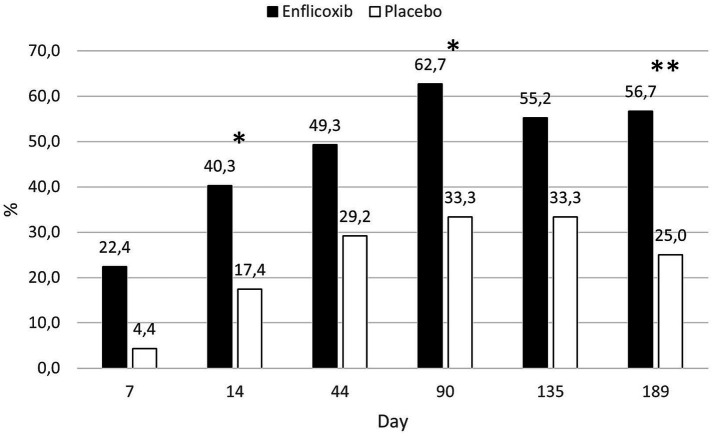
Percentage of CBPI responders in each treatment group and time point during the study. Asterisks indicate superiority vs. placebo (*p < 0.05, **p < 0.01).

The response to treatment according to the owner assessment (CBPI) was evident in the enflicoxib treated group from the first week of treatment, reaching a plateau of 50–60% of responders throughout the follow up period. In the placebo group, the percentage of responders increased slowly during the first weeks reaching a plateau of around 30% during most of the follow up period. However, at the last visit, the percentage of responders decreased to 25%. Statistically significant differences in the percentage of responders after treatment with enflicoxib vs. placebo were reached on Days 14 and 90 (*p* < 0.05) and 189 (*p* < 0.01). Figure 4 shows the treatment response rate according to the CBPI throughout the study.

**Figure 5:**
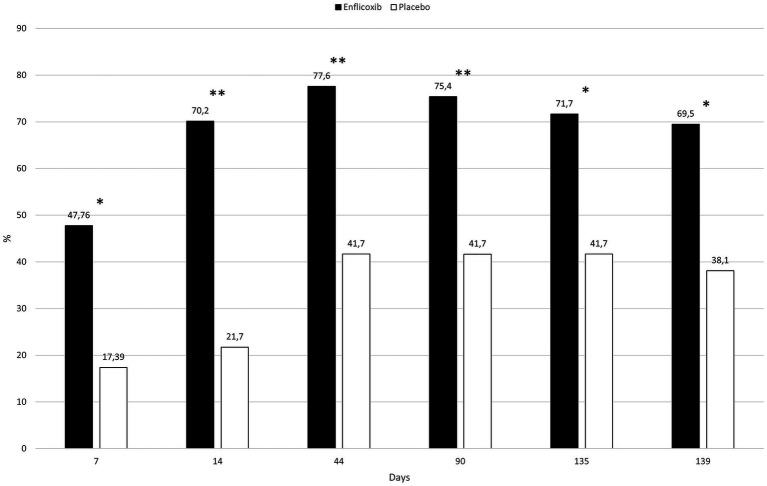
Evolution of the percentage of dogs that improved at least one category on the owner’s perception of quality of life in each treatment group and time point compared to D0. Asterisks indicate superiority vs. placebo (*p < 0.05, **p < 0.01).

The overall owner impression of the dog’s QoL improved in around 70% of the dogs treated with enflicoxib throughout the study, whereas in the placebo group this percentage increased slowly to a 40% of dogs on Day 44 and remained stable, with no further improvement, until the end of the study. The differences between groups were statistically significant throughout the study starting at the first week of treatment (*p* < 0.05). Figure 5 depicts the percentages of dogs improving in the owner’s perception of the dog’s QoL in each treatment group throughout the study.

### 3.2 Safety evaluation

The results of the main hematological, biochemical and urine parameters are summarized in Tables 2, 3. The values obtained for all the hematological and biochemical parameters were generally within the reference ranges of the laboratory of analyses and no statistically significant differences were observed in any parameter, except for a slight increase in average urea values in the enflicoxib group on Day 189 (8.3 vs. 7.5 mmol/L in the treated and control group respectively; Laboratory reference range: 3.5–10 mmol/L).

**Table 2:** Selected hematology parameters mean values (SD) for dogs in both treatment groups.

Treatment group		Enflicoxib	*n*	Placebo	*n*	*p* ^†^
Red blood cell count	Basal	7.2 (1.0)	81	7.3 (0.7)	26	ns
(×10^12^/L) (RBC)	Day 44 (1.5 months)	7.2 (1.1)	75	7.6 (0.8)	26	ns
	Day 189 (6 months)	7.0 (0.9)	64	7.3 (1.1)	24	ns
Reticulocytes	Basal	73.0 (42)	42	74.5 (31)	12	ns
(×10^3^/ul) (Retic)	Day 44 (1.5 months)	75.2 (37)	39	73.4 (32)	14	ns
	Day 189 (6 months)	59 (30)	28	77.6 (25)	13	ns
Hemoglobin (g/L)	Basal	164 (20)	81	166 (14)	26	ns
(Hb)	Day 44 (1.5 months)	163 (20)	75	172 (14)	26	ns
	Day 189 (6 months)	159 (18)	64	164 (23)	24	ns
Hematocrit (%)	Basal	50.1 (7.7)	81	51.6 (5.7)	26	ns
(Hct)	Day 44 (1.5 months)	49.7 (7.5)	75	52.9 (5.3)	26	ns
	Day 189 (6 months)	49.5 (6.6)	64	51.7 (7.8)	24	ns
Platelets (×10^9^/L)	Basal	292 (123)	81	320 (157)	26	ns
(Hct)	Day 44 (1.5 months)	295 (131)	75	356 (178)	26	ns
	Day 189 (6 months)	274 (93)	64	292 (170)	24	ns

**Table 3:** Selected biochemistry parameters mean values (SD) for dogs in both treatment groups.

Treatment group		Enflicoxib	*n*	Placebo	*n*	*p* ^†^
Alkaline phosphatase (U/L)	Basal	107 (96)	81	97 (65)	26	ns
(ALP)	Day 44 (1.5 months)	95 (73)	75	111 (88)	26	ns
	Day 189 (6 months)	84 (52)	64	80 (47)	24	ns
Alanine aminotransferase (U/L)	Basal	52 (31)	83	52 (44)	27	ns
(ALT)	Day 44 (1.5 months)	48 (31)	81	44 (30)	28	ns
	Day 189 (6 months)	41 (20)	83	42 (24)	27	ns
Aspartate aminotransferase (U/L)	Basal	42 (26)	83	36 (11)	27	ns
(AST)	Day 44 (1.5 months)	43 (40)	81	35 (11)	28	ns
	Day 189 (6 months)	39 (41)	83	35 (10)	27	ns
Urea (mmol/L)	Basal	6.3 (2.5)	81	6.8 (3.0)	26	ns
	Day 44 (1.5 months)	7.8 (2.9)	75	7.1 (2.5)	26	ns
	Day 189 (6 months)	8.3 (4.2)	65	7.5 (6.4)	24	*
Creatinine (μmol/L)	Basal	97 (28)	81	90 (21)	26	ns
	Day 44 (1.5 months)	101 (42)	75	96 (29)	26	ns
	Day 189 (6 months)	109 (62)	65	97 (31)	24	ns
Total Protein (g/L)	Basal	73 (7)	81	73 (7)	26	ns
	Day 44 (1.5 months)	69 (8)	75	71 (8)	26	ns
	Day 189 (6 months)	66 (10)	65	67 (6)	24	ns
Albumin (g/L)	Basal	32 (6)	81	33 (5)	26	ns
	Day 44 (1.5 months)	31 (5)	75	32 (5)	26	ns
	Day 189 (6 months)	31 (6)	65	31 (5)	24	ns
Cholesterol (mmol/L)	Basal	5.4 (1.7)	81	5.2 (1.5)	26	ns
	Day 44 (1.5 months)	5.5 (1.5)	75	5.5 (1.3)	26	ns
	Day 189 (6 months)	5.3 (1.3)	64	4.9 (1.3)	23	ns
Glucose (mmol/L)	Basal	4.2 (1.5)	81	4.4 (1.4)	26	ns
	Day 44 (1.5 months)	4.2 (1.3)	75	4.5 (1.0)	26	ns
	Day 189 (6 months)	4.3 (1.4)	65	4.7 (0.8)	24	ns
Urinary specific gravity	Basal	1.03 (0.01)	80	1.02 (0.01)	25	ns
	Day 44 (1.5 months)	1.04 (0.03)	75	1.03 (0.01)	25	ns
	Day 189 (6 months)	1.05 (0.06)	60	1.03 (0.01)	23	ns
Urinary pH	Basal	7.0 (0.8)	81	6.9 (0.9)	25	ns
	Day 44 (1.5 months)	7.0 (0.8)	75	7.0 (0.8)	25	ns
	Day 189 (6 months)	6.9 (0.8)	60	6.9 (0.9)	23	ns

ns: *p* > 0.05, *p < 0.05, ** < 0.01.

**Figure 6:**
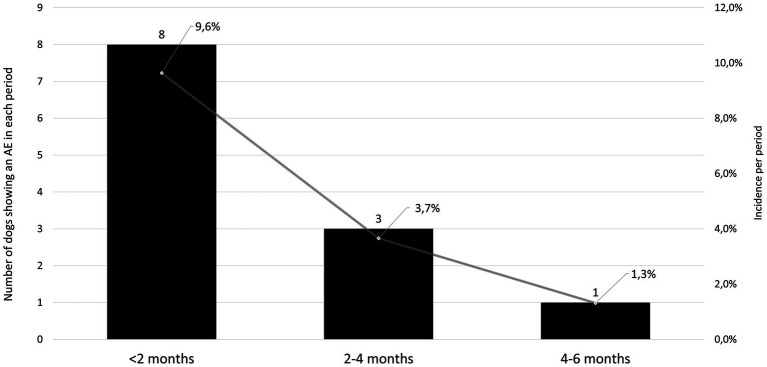
Number (solid bars, left axis) and incidence (solid line, right axis) of adverse events (AE) in the treated group at different periods during the study showing a decreasing incidence over time.

From the ITT population of 109 dogs, a total of 22 AEs in 19 dogs were reported during the study. Nineteen of these AEs occurred in 16 of the 83 dogs treated with enflicoxib and three AE in 3 of the 26 dogs included in the placebo group. When the AEs were classified according to the ABON system for causality assessment, 9 reported AEs were classified as “N,” and therefore, unlikely to be product related. All other 13 AEs (twelve in dogs treated with enflicoxib and one in a dog receiving placebo) fell into categories “A,” “B,” or” “O”, as a causal relation to treatment could not be ruled out (incidence 14.4 and 3.8% respectively, *p* = 0.267). From these AEs, the veterinarian classified the reaction in 7 animals treated with enflicoxib and one in the placebo group as being “drug related” (incidence 8.4 and 3.8%, respectively. *p* = 0.725). The incidence of AEs in the enflicoxib group decreased from 9.6% during the first 2 months of treatment to a 1.3% at the end of the study, as shown in Figure 6.

The main digestive tract disorders were diarrhea or vomiting (7.2% in the enflicoxib group and 3.8% in the placebo group. *p* = 0.986). Most cases were transient and of mild to moderate nature and were not considered to be a concern to discontinue treatment. A description of the reported cases showing gastrointestinal disorders is included in Table 4.

**Table 4:** Digestive tract disorders reported as AE, classified as A, B or O.

Case #	Group	Description
H-02-02	Enflicoxib	Ten years old mixed breed female. After 3 months of treatment, it shows vomiting and treated symptomatically with famotidine and recovers completely without discontinuing enflicoxib treatment.
H-04-01	Enflicoxib	Female Leonberger female of 10 years old. After seven doses of treatment the dog starts showing apathy, appetite loss and laboratory results show macrocytic hypochromic regenerative anemia compatible with GI bleeding and FOB+. The dog was withdrawn from the study, treated with famotidine, sucralfate and catosal, and recovered completely.
H-02-14	Enflicoxib	Thirteen years old Dachshund male. After four doses of treatment the dog shows vomiting and treated symptomatically with famotidine and recovers completely without discontinuing enflicoxib treatment.
H-01-04	Enflicoxib	Nine years old male German Shepard. After 14 doses the dog owner saw blood in the feces. The dog was withdrawn (due to also worsening of OA symptoms, the dog could not stand up) and symptomatic treatment with famotidine and sucralfate was started and the dog recovered completely after five days.
P-02-06	Enflicoxib	Eleven years old mixed breed male. After 7 doses the dog shows bloody diarrhea. Treated with probiotics. The dog was withdrawn by owner decision but recovered completely.
P-02-08	Enflicoxib	Ten years old English Pointer male. After 6 doses of treatment the dog shows moderate diarrhea for less than 48 h and recovered completely without treatment. Enflicoxib treatment was not discontinued until the end of the study.
H-02-08	Placebo	Ten years old Jack Terrier male. After 3 months of treatment with placebo the dog shows diarrhea and vomiting. Treated with probiotics, sucralfate, famotidine, and B vitamins complex and recovered completely. The dog continued in the study.

Other non-serious AE reported in the enflicoxib group with lower incidence included one case with acute pancreatitis, one dog with altered liver enzymes and another dog with reported lack of expected efficacy.

Three AEs in three animals treated with enflicoxib were classified as serious: Case H-02-07 was an entire 9-year-old male of 41.2 kg bw purebred American Bulldog with no comorbidities or concomitant medications. On Day 44 the laboratory results showed altered hematology parameters compatible with anemia and increased liver values. The dog was withdrawn and started treatment. Over 1 month later, the dog was not eating or drinking and did not stand up, laboratory results were worse, and the dog was euthanized. According to the ABON system the case was classified as possibly (B) related to treatment but, not attributed to an excessive exposure to the product since blood levels of enflicoxib or its metabolite on Day 44 were in the expected range. Case H-04-10 was a castrated mixed breed female of 21 kg bw and 11 years old with no comorbidities or concomitant medications. On Day 14, the dog was found dead with no previous signs of disease. No valid blood sample to test product levels was obtained. Necropsy and histopathology were performed but were inconclusive. The case was classified as ABON=B although, according to the veterinarian the most probable cause of death was a septic shock. Finally, case H-02-10 was a castrated mixed breed female of 37 kg bw and 8 years old. After 5 months of treatment, it showed severe vomiting and abdominal pain, anorexia, and difficulty to move. Blood analysis revealed acute pancreatitis. The dog deteriorated and had several seizures and was euthanized due to its poor condition. Blood levels of enflicoxib or its metabolite on Day 44 were in the expected range. Pancreatitis is not expected to be an adverse effect of NSAIDs but, since the effects of treatment cannot be completely ruled out, the case was classified as inconclusive (ABON=O).

## 4 Discussion

This is the first clinical study assessing the safety and efficacy of a 6 months treatment of enflicoxib in dogs suffering from OA.

Two previous clinical trials had already demonstrated the clinical efficacy and the good safety profile of enflicoxib treatment in the target population over a period of 6 weeks (8, 9). However, as OA is a chronic process that normally requires long-term treatments, very often in geriatric dogs, it is not only the short-term efficacy that drives the selection of the most adequate treatment, but also the good tolerance in chronic treatments in such population. Therefore, this new study provides answers in confirming both, the efficacy, and the safety of enflicoxib, in a treatment duration of 6 months, thus allowing a fully informed decision to be made by veterinarians regarding the use of the product as a long-term therapy.

In this study, basal CSS was the only variable that was not balanced between treatments (*p* < 0.05) on Day 0. However, the main efficacy parameter (CSS responders) is already defined considering each dog basal value. Therefore, this initial unbalance is not considered to have an influence on the outcome of the study.

The results of this study show that there was an overall improvement in dogs treated with enflicoxib. Most dogs had improved clinical parameters in the first veterinary assessment on Day 44 and the efficacy was sustained until the end of the 6 months follow up period, with clear statistical superiority over the placebo group. The high placebo effect observed in the first clinical control tends to diminish with time and, at the end of treatment, treatment response in the placebo group had decreased by half, and average CSS scores were only slightly lower than the basal ones. The high placebo effect observed in this study is in line with what has previously been described in similar studies (8, 9, 21–24). Owner assessments confirmed the long-term efficacy with significant treatment response superiority over placebo from the second week of treatment. The owner perception of QoL of the dogs showed statistically significant differences vs. placebo throughout the study, with approximately 70% of dogs improving their wellbeing from the assessment on Day 14 until the end of treatment. The overall results of this study show that the long-term efficacy profile of enflicoxib follows a similar pattern as in the published clinical studies of shorter duration, rapidly achieving efficacy and maintaining it for as long as treatment is continued.

Only one dog in the control group had to be withdrawn due to lack of efficacy. This is probably due to the small size of this group, the high placebo effect observed, and a lack of a fixed rule for withdrawal, that was left to the veterinarian or owner decision. From the scientific point of view, it is more robust to compare efficacy and safety to placebo control groups (10). However, in this study, the negative control group was reduced to the minimum statistically meaningful as a long-term placebo treatment for a condition that is known to be painful such as OA has ethical implications. Still, as estimated in the sample size calculation, this group size was large enough to demonstrate statistically significant differences compared to the enflicoxib group.

Regarding safety, in a previous target animal safety study (11), enflicoxib showed a wide safety margin when young healthy Beagle dogs were treated at 5 and 3 times the therapeutic dose for 3 and up to 7 months, respectively. In a more realistic scenario, in the two previous clinical studies (8, 9) a good safety profile was observed in the target population of dogs with OA that could be considered as a geriatric population with other co-morbidities and receiving concomitant medications. However, as these clinical studies did not report the evolution of hematological, biochemical or urine parameters, it was not known whether the general conclusions of the target animal safety study in Beagle dogs would directly apply to the real conditions of use of the product in this especially sensitive target population.

In this study, the hematological, biochemical and urine parameters of the dogs treated with enflicoxib showed no differences compared to the placebo group, with no indication of hepatic or kidney involvement. Only average urea concentrations showed a slight increase in the treated group that was statistically significant compared to the placebo group in the last sampling on Day 189 only. However, these values were largely within the laboratory reference range and were not accompanied by any other alterations. A rise in urea concentrations could be related to renal dysfunction but could also be induced by other factors such as a high protein diet, gastrointestinal bleeding, or subclinical dehydration with age (25).

Changes in serum creatinine concentrations suggesting some degree of kidney involvement are a common finding in studies with nonspecific NSAIDs (26–29) and with selective COX-2 inhibitors (30–33). However, in this study, no increase in creatinine was observed and urine analysis remained within normal limits throughout the study. In fact, the above-mentioned increased urea levels follow a pattern like in the overdose target animal safety study (11), where no histopathological changes in the kidneys or the gastrointestinal tract were observed after a seven-month treatment. This makes it unlikely that a decreased renal function or a gastrointestinal damage were the cause of the increased urea values.

Any given dog showing any clinical sign or clinically relevant alterations of hematological, biochemical or urine parameters was considered as an AE and assessed for causality related to treatment. The incidence and type of AEs possibly related to enflicoxib administration was similar to the previous clinical studies with a six-week treatment duration [14.4, 19.7, and 25.6% incidence of AEs classified as A, B, or O were found in this study and in Salichs et al. (8, 9) respectively]. In this study, the extended duration of the treatment did not translate in a higher incidence of AE. Moreover, most of the AE occurred in the first 2 months, and the incidence, in fact, clearly decreased in the following months.

As expected, mild digestive alterations were the most frequently reported in all three studies, although in this long-term study only 3 dogs discontinued the treatment due to a gastrointestinal problem. These incidences are similar to or lower than those reported in similar studies with other NSAIDs (15, 33–39) or piprants (21), and can be considered typical for a population of older dogs with OA and incidental comorbidities treated with this type of medication (37). On the other hand, no similar signs to those observed in the three serious AEs reported in this study, have been described in the previous clinical or safety studies with enflicoxib (8, 9, 11).

With the results of the present study, safety seems to be confirmed, adding a longer-term perspective not only in efficacy, but also in safety, as dogs received up to 27 doses of the product, covering a period of 6 months. This is particularly important since side effects of NSAID treatment are often a limiting factor for veterinarians when considering prescribing an NSAID for a long period of time. However, it is still recommended that treatments are accompanied by regular monitoring of clinical and clinicopathological evaluations.

The similarity of the incidences observed in the three clinical studies performed with enflicoxib and in comparison to others, indicates that treatment duration does not increase the risk of AEs, confirming the observations of Innes et al. (10). On the other hand, it is known that, when left untreated, OA can progress to a severe debilitating disease with significant functional impairment and pain sensitization that could lead to chronic maladaptive pain, so, arguably, the benefits of a chronic NSAID dose regimen outweighs the perceived risks (10).

Indeed, it is currently accepted that there is a clinical benefit of a sustained pain control with long-term NSAID therapy, as it would lead to a reduction in central sensitization and a concomitant progressive reduction in the pain perceived by the osteoarthritic dog (10). Moreover, as central sensitization can drive the progression of disease in the periphery (joints), a downward modulation of central sensitization would result in decreased joint pathology (3, 10). This study supports the chronic administration of enflicoxib for continued improvement in the multimodal management of osteoarthritic pain in dogs as opposed to short treatment cycles or treatments “on demand” to cover flare-ups. This approach would better preserve the dog’s wellbeing as subtle or intermittent behavioral alterations due to orthopedic changes may go undetected if owners do not associate the changes with evidence of their dog being in pain (40). As a result, veterinarians are frequently not consulted until the dog’s behavioral changes or impaired activity are more marked and have already caused unnecessary pain and possibly central or peripheral sensitization (41).

On the other hand, in long-term treatments, owner compliance may be impaired if products need too frequent administration, and this may obviously have negative effects in the efficacy of pain control and, consequently, in the wellbeing of the dog (42). In addition, geriatric dogs usually need simultaneous administration of other medications to treat concomitant pathologies which further contribute to pet owner burden (37). In this sense, enflicoxib has a dose regime of weekly dosing intervals which offers clear advantages from the treatment compliance point of view, contributing to its real efficacy in normal practice.

## 5 Conclusion

Long-term enflicoxib administration demonstrated a sustained level of efficacy and an adequate safety profile, which showed to be advantageous for improving the QoL of client owned dogs with naturally occurring OA.

## Data availability statement

The raw data supporting the conclusions of this article will be made available by the authors, without undue reservation.

## Ethics statement

The requirement of ethical approval was waived by Portuguese and Hungarian regulatory authorities: DGAV (Direção Geral de Alimentação e veterinária) and NEBH (Nemzeti Élelmiszerlánc-biztonsági Hivatal), with authorization numbers 82/ECVPT/2020 and 5300/3265-2/2020 respectively for the studies involving animals because clinical studies on veterinary pharmaceutical products are assessed and authorized by the regulatory authorities according to pharmaceutical legislation. The studies were conducted in accordance with the local legislation and institutional requirements. Written informed consent was obtained from the owners for the participation of their animals in this study.

## Author contributions

JH: Conceptualization, Methodology, Writing – original draft. MO: Data curation, Formal analysis, Software, Writing – review & editing. SR: Data curation, Investigation, Project administration, Writing – review & editing. MS: Conceptualization, Supervision, Writing – original draft.
