# Supplementary material for: A blinded, randomized and controlled multicenter field study investigating the safety and efficacy of long-term use of enflicoxib in the treatment of naturally occurring osteoarthritis in client-owned dogs
Source: Front Vet Sci. 2024 Feb 23;11:1349901. doi: 10.3389/fvets.2024.1349901 (PMC10995975; doi:10.3389/fvets.2024.1349901)
Supplement: Supplementary file 1 [file Data_Sheet_1.PDF]

Supplementary material:

Grading for categories of Veterinary Assessment of pain and lameness (CSS)

Lameness/Weight Bearing

0 – Clinically Normal. Stands, walks and trots normally with equal weight-bearing on all limbs.

1 – Mild. Stands and walks normally. Bears slightly less weight on affected limb when trotting.

2 – Moderate. Stands normally. Bears slightly less weight on affected limb when walking.

3 – Severe. Abnormal posture when standing due to unequal weight-bearing on limbs. Bears much less weight on affected limb(s) when walking.

4 – Nearly incapacitated. Non-weight bearing on affected limb(s) at rest or when walking or may be reluctant to rise and unwilling to walk more than five strides if more than one limb is affected.

Pain on Palpation/Manipulation of Joint(s)

0 – Clinically Normal. No pain evident in the normal range of joint movement.

1 – Mild. Mild pain evident on palpation and/or manipulation of affected joint(s): e.g. animal appears tense.

2 – Moderate. Moderate pain evident on palpation and/or manipulation of affected joint(s): e.g. animal turns head in recognition.

3 – Marked. Pronounced pain evident on palpation and/or manipulation of affected joint(s): e.g. animal turns and pulls limb(s) away.

4 – Severe. Animal vocalises or becomes aggressive on palpation and/or manipulation of affected joint(s).

General Musculoskeletal Condition

The grade assigned for this assessment should reflect the overall range of severity and clinical presentation of the disease condition affecting the animal during the assessment.

0 – Clinically Normal. Does not demonstrate clinical signs associated with the inflammation and/or pain of osteoarthritis.

1 – Mild. Gait and weight-bearing infrequently affected. Overall mobility not affected. Rarely, if ever, painful. The clinical signs may be intermittent.

2 – Moderate. Gait and weight-bearing may be affected. May present lame at times. Overall mobility may be affected at times, but not severely. Infrequently painful.

3 – Severe. Gait and weight-bearing often affected. Overall mobility may be severely or frequently hindered. Frequently painful. Clinical signs are frequent.

4 – Nearly incapacitated. May be unwilling to bear weight or walk at times. Overall mobility severely compromised. Generally painful. Clinical signs are continually evident.
